# miR-146b suppresses LPS-induced M1 macrophage polarization via inhibiting the FGL2-activated NF-κB/MAPK signaling pathway in inflammatory bowel disease

**DOI:** 10.1016/j.clinsp.2022.100069

**Published:** 2022-06-21

**Authors:** Yang Pan, Dan Wang, Fan Liu

**Affiliations:** a Department of Laboratory Medicine, Wuhan Children's Hospital (Wuhan Maternal and Child Healthcare Hospital), Tongji Medical College, Huazhong University of Science & Technology, China; b Digestive Department, Wuhan Children's Hospital (Wuhan Maternal and Child Healthcare Hospital), Tongji Medical College, Huazhong University of Science & Technology, China; c Department of Rheumatology and Immunology, Wuhan Children's Hospital (Wuhan Maternal and Child Healthcare Hospital), Tongji Medical College, Huazhong University of Science & Technology, China

**Keywords:** Inflammatory bowel disease, miR-146b, FGL2, Inflammation, M1 Macrophage polarization

## Abstract

**Objectives:**

M1 macrophage polarization and phenotype in Inflammatory Bowel Disease (IBD) are common biological responses.

**Method:**

Herein, IBD mice models were constructed and macrophages were derived.

**Results:**

It was discovered that microRNA-146b (miR-146b) was downregulated in IBD mice and Lipopolysaccharide (LPS)-induced macrophages. Moreover, the inhibitory role of overexpressed miR-146b in reducing the inflammation level and blocking M1 macrophage polarization was confirmed. Further investigation indicated that Fibrinogen Like 2 (FGL2) acted as the target gene of miR-146b, and FGL2 mediated activation of NLRP3, NF-κB-p65, and p38-MAPK. More importantly, it was validated that miR-146b could ameliorate inflammatory phenotype and prevent M1 macrophage polarization via inhibiting FGL2 *in vitro*, and miR-146b overexpression alleviated the intestinal injury of IBD mice *in vivo*.

**Conclusions:**

Overall, it is potential to use miR-146b for the amelioration of IBD.

## Highlights

• miR-146b was downregulated in Inflammatory Bowel Disease (IBD) mice and LPS-induced macrophages.
• Fibrinogen Like 2 (FGL2) was identified as the target gene of miR-146b.
• miR-146b ameliorated the inflammation and blocked M1 macrophage polarization via inhibiting FGL2.
• miR-146b ameliorated the symptoms and pathological injury of IBD via inhibiting FGL2.

## Introduction

Inflammatory Bowel Disease (IBD) is a non-specific chronic intestinal inflammatory disease that mainly affects the ileum, rectum, and colon [1]. Currently, IBD is classified into Ulcerative Colitis (UC) and Crohn's Disease(CD) [2]. Among them, the former mainly damages the colon and rectum [3], while the latter has a higher incidence at the end of the small intestine and colon [4]. Clinically, IBD is usually manifested as abdominal pain, diarrhea, weight loss, and even bloody stools [5]. However, it is difficult to cure IBD, which is characterized by a high recurrence rate [6]. Therefore, it is urgent to find and investigate targets for the prediction and alleviation of IBD.

Macrophages, also known as histocytes, are differentiated from monocytes in the blood [7]. According to different activation states and functions, macrophages can be classified into M1 type (classically activated macrophages) and M2 type (alternatively activated macrophages) [8,9]. Among them, M1-type macrophages secrete pro-inflammatory cytokines and chemokines, and perform the function of immune surveillance [10]. Reversely, M2 type macrophages can down-regulate the immune response by secreting inhibitory cytokines like IL-10 or TGF-B [10,11]. Interestingly, pro-inflammatory cytokines can induce polarization of M1 type macrophages to M2, thereby ameliorating the inflammation response [10,12].

MicroRNAs have been identified as a type of small molecular RNA, which play an indispensable role in modulating IBD progression. According to previous evidence, MicroRNAs such as microRNA-155[13], miR-142-3p[14] and MiR-223[15] affected inflammation in IBD. More specifically, Guo et al [16] disclosed that miRNA-29c-3p could deteriorate the inflammation level in Ulcerative Colitis through mediating leukemia inhibitory factor, Li et al. demonstrated that miR-155 accelerated the intestinal fibrosis in IBD through modulating HBP1/Wnt/β-catenin signaling pathway. Additionally, Zhou et al [17] determined miR-16-5p and miR-21-5p in feces as the markers predicting the inflammation level of IBD. Interestingly, as a member of microRNAs, miR-146b has been proved to ameliorate epithelial barrier function in IBD through stimulating nuclear factor-Κb [18], while the other miR-146b-related mechanisms are involved in IBD and polarization of M1 macrophages remain unknown.

Thus, the authors investigated the expression of miR-146b and FGL2 in IBD mice and LPS-induced macrophages, and it was revealed that the two were correlated with each other. Moreover, the functional regulation of miR-146b/FGL2 in inflammation and polarization of M1 macrophages was investigated, which may be promising markers for the diagnosis of IBD.

## Materials and methods

### Construction of IBD mice models

For the establishment of IBD mice models, BALB/c mice (male, 6–8 weeks) were used and divided into Normal group (n = 6), and dextran sodium sulfate (DSS)-induced IBD group (IBD, IBD+ NC-agomir, IBD+ miR-146b-agomir, n = 6). Specifically, mice in the IBD groups were fed with drinking water containing 2.5% DSS for the first 5 days, and then the mice in IBD+NC-agomir and IBD+miR-146b-agomir groups were additionally injected with miR-146b agomir and NC-agomir through the tail vein for the following 5 days. The weight and Disease Activity Index (DAI) score was monitored, which was in accordance with the previous report [19]. In addition, the length calculation and HE-staining of the colon were performed at day 14, and the histological score was determined as described in the previous reference [20]. All applicable international, national, and/or institutional guidelines for the care and use of animals were followed. This study was approved by Wuhan Children's Hospital.

### Macrophage isolation and treatment

According to the previous report [21], PBS was utilized to flush the peritoneal cavity for the isolation of macrophages. Then the collected cells were added to 12-well plates covered by Roswell Park Memorial Institute medium with 10% FBS and 1% penicillin; 100 ng/mL Lipopolysaccharide (LPS) was then added.

To finish transient transfection, miR-146b-mimics, NC-146b-mimics, miR-146b-inhibitors, NC-inhibitors, si-FGL2-1, and si-FGL2-2 were designed and supplied by GeneChem Corporation (Shanghai, China) (Supplementary Table 1).

### ELISA

The Mouse TNF alpha ELISA Kit (ab208348), Mouse IFN gamma ELISA Kit (ab282874), Mouse IL-23 ELISA Kit (ab119545), and Mouse IL-10 ELISA Kit (ab255729) were used for the determination of inflammation-related factors in the colon tissues and macrophages.

### RIP

For confirmation of the interaction between miR-146b and FGL2, EZ-Magna RIP Kit (Millipore) was used in this RIP assay. Briefly, after lysis of macrophages by RIP lysis buffer, the lysed samples were mixed with RIP buffer harboring Anti-Ago2 or Anti-IgG conjugated magnetic beads, and the immunoprecipitated RNAs of FGL2 were quantified utilizing q-PCR.

### Dual Luciferase reporter assay

Dual Luciferase reporter assay was performed to investigate the association between miR-146b and FGL2. In brief, miR-146b mimics or NC mimics were co-transfected with FGL2-MUT or FGL2-WT into 293T cells, followed by quantification of the luciferase activity of FGL2 using Dual-Luciferase Reporter Assay (Promega, Madison, United States).

### Western blotting

Before quantification of proteins, RIPA buffer was utilized to dissolute colon tissues and macrophages for protein extraction. After pelleting detergent-insoluble cell debris, BCA protein assay kit was applied to quantify proteins. Then, 6% SDS-PAGE was utilized for the separation of protein samples and the isolated proteins were transferred to PVDF membranes, followed by blocking in a solution containing 5% non-fat dry milk for 1h. Subsequently, these PVDF membranes were cultivated overnight at 4°C with primary antibodies, FGL2 (ab198029, 1/450), NLRP3 (ab263899, 1/1000), NF-κB-p65 (ab246347, 1/1000), p38-MAPK (ab170099, 1/2000), p-NF-κB-p65 (ab239882, 1/1000), p-p38-MAPK (ab60999, 1/500), NOS2 (ab178945, 1/1000), TLR2 (ab209217, 1/1000), CD86 (ab269593, 1/1000), and they were mixed with secondary antibody conjugated by horseradish peroxidase at room temperature. ImageQuant LAS 500 imager and WesternBright ECL chemiluminescent substrate (Advansta, Inc.) were respectively used for the quantification of proteins and visualization of Western blots.

### qRT-PCR

To test the RNA expression in colon tissues and macrophages, all RNA samples were isolated by HiPure Total RNA Mini Kit and RNAiso-Plus (TAKARA, China), and NanoDrop was introduced for the quantification. Then ReverTra Ace qPCR RT Kit (Toyobo, China) was used for reverse transcription. iQTM SYBR® Green Supermix (Bio-Rad) was employed for the estimation of RNA expression. All primer sequences used in RNA analysis are elaborated in Supplementary Table 2.

### Statistical analysis

In this paper, all the experimental data were expressed as mean ± Standard Deviation (SD) and were analyzed by GraphPad Prism 6.0. The differences between the two groups were evaluated using Student's *t*-test, while the differences among several groups were assessed by ANOVA; p < 0.05 indicated a significant difference.

## Results

### miR-146b is lowly expressed in IBD mice

**Fig. 1:**
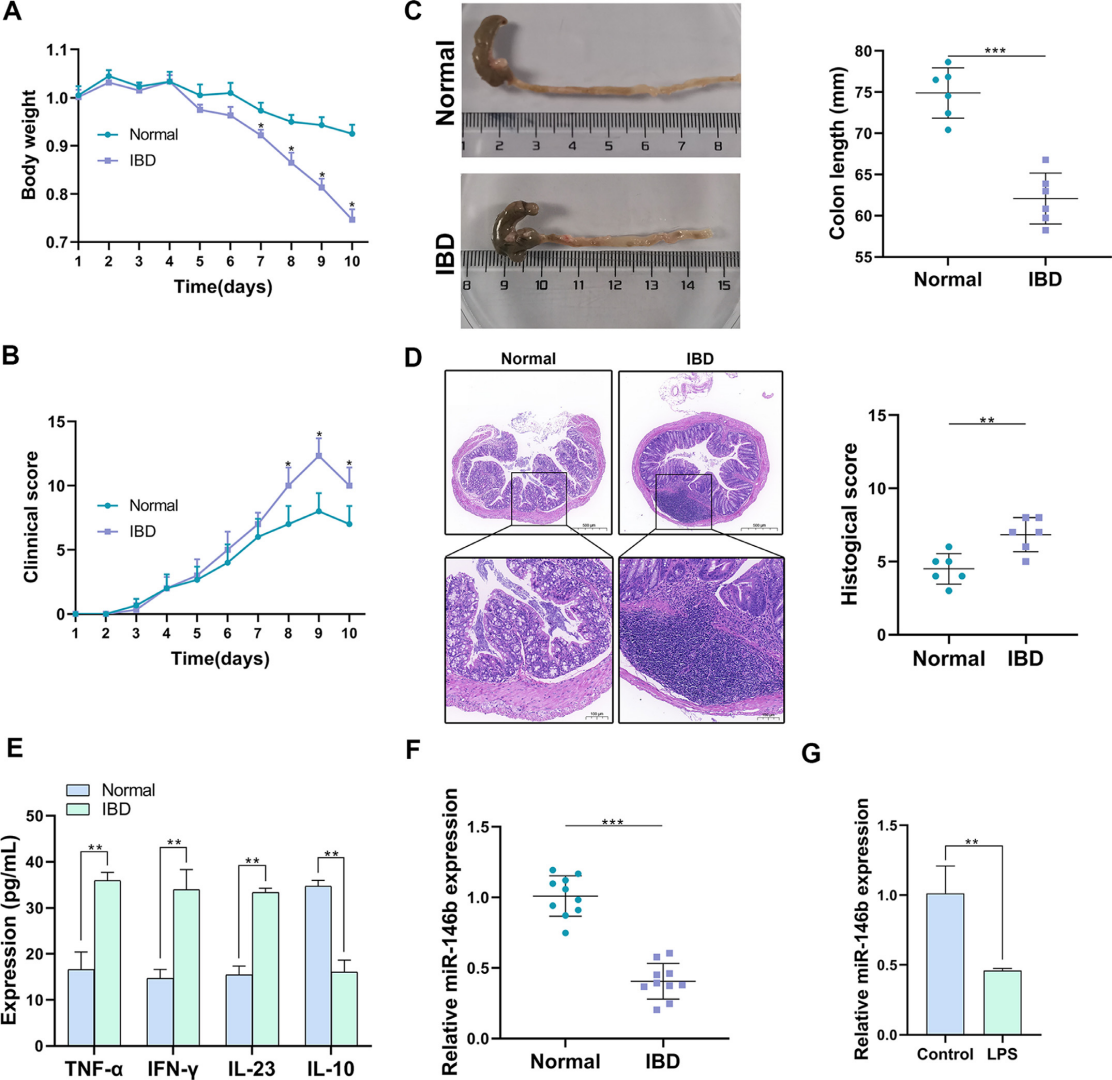
miR-146b is lowly expressed in IBD mice models. The body weight (A) and DAI scores (B) of IBD mice during the DSS induction. The changes of colon were monitored and the length was measured. (C) colon length and the pathological changes. (D) HE staining and histological scores of colon tissues. (E and F) ELISA Kits were used for the determination of inflammation-related factors (E) and the expression of miR-146 in IBD mice (F). LPS-induced macrophages (G) was determined by q-PCR.

To confirm the expression of miR-146b in IBD, the authors first established IBD mice models using 2.5% DSS. Obviously, during the period of DSS induction, the body weight of mice in the IBD group was much lighter than that in the Normal group (Fig. 1A), while the comprehensive Disease Activity Index (DAI) in IBD mice was considerably elevated (Fig. 1B). In addition, it was noticed that DSS significantly reduced colon length (Fig. 1C), while aggravated the inflammatory cell infiltration, which was indicated by the higher histological score (Fig. 1D). Furthermore, the inflammation-related factors were determined using ELISA Kits, and it was found that the pro-inflammatory cytokines like TNF-α, IFN-γ, and IL-23 were upregulated in colon tissues of IBD mice, while the anti-inflammatory cytokine IL-10 was reduced (Fig. 1E). Based on the successful construction of IBD mice models, the expression of miR-146b in colon tissues was examined, and it was found that miR-146 was lowly expressed in IBD mice, compared with that in Normal mice (Fig. 1F). For further confirmation, macrophages were isolated from IBD mice, and it was found that miR-146 was downregulated in LPS-induced macrophages (Fig. 1G). In summary, the present results validated the downregulation of miR-146b in IBD mice, indicating that miR-146 may be involved in IBD development.

### miR-146b overexpression alleviates inflammation response and blocks M1 polarization of macrophages

**Fig. 2:**
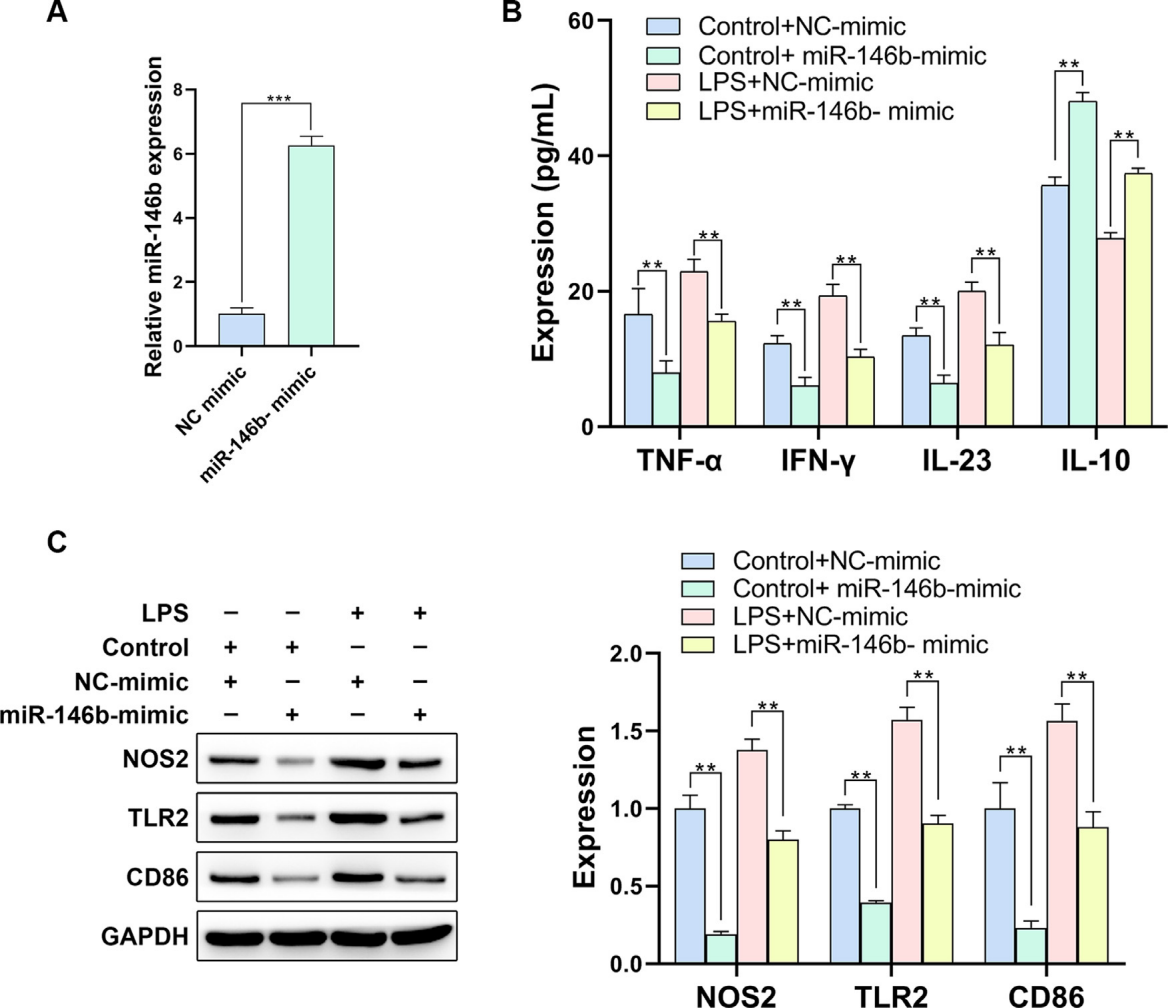
miR-146b overexpression alleviates inflammation responses and blocks M1 polarization in macrophages. (A) The transfection efficacy of miR-146b overexpression was examined by q-PCR. (B) The levels of inflammation-related factors were determined by ELISA assay. (C) M1 macrophage polarization-related indicators were examined by Western blotting.

To investigate whether miR-146b plays a functional role in IBD-related inflammation phenotype, macrophages were transfected with miR-146b mimics (Fig. 2A). ELISA assay displayed that LPS induction resulted in the obvious rise of TNF-α, IFN-γ, and IL-23, which could be blocked by miR-146b overexpression. Meanwhile, the LPS-induced decline of IL-10 was rescued by miR-146b (Fig. 2B). Furthermore, the authors examined the M1 macrophage polarization-related indicators, and it was observed that upregulated miR-146b acted as an inhibitor in LPS-induced elevation of NOS2, TLR2, and CD86 expression (Fig. 2C). These findings confirmed the suppressive effect of miR-146b upon LPS-induced inflammation phenotype and M1 macrophage polarization.

### miR-146b inhibits FGL2 in IBD-derived macrophages

**Fig. 3:**
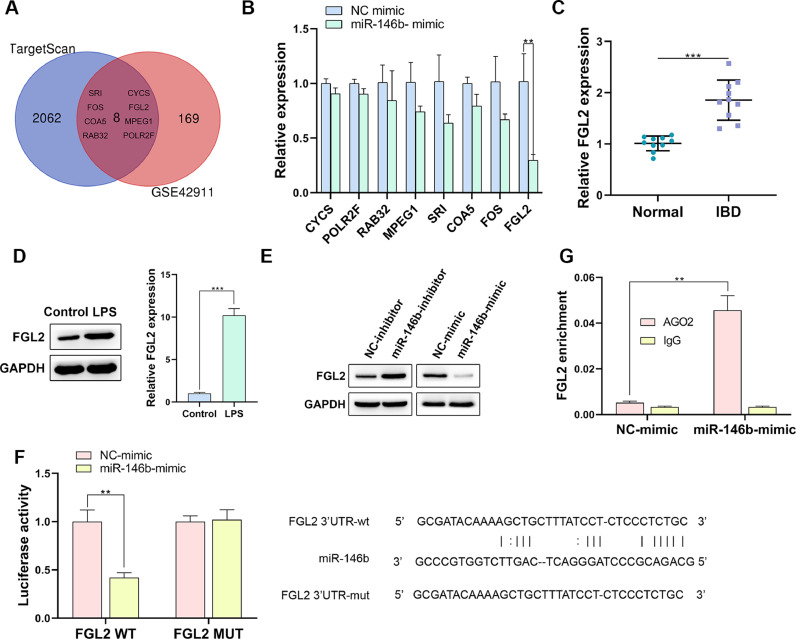
miR-146b inhibits FGL2 in IBD-derived macrophages. (A) TargetScan database and GSE42911 dataset were used to screen the target gene of miR-146b. (B) The expression of intersected genes in miR-146b-overexpressed cells was examined by q-PCR (C and D) The expression of FGL2 in IBD mice (C) and LPS-induced macrophages (D) was determined by q-PCR and Western blotting. (E) FGL2 expression in miR-146b-overexpressed or miR-146b-depleted macrophages was determined by Western blotting. (F and G) The correlation between miR-146b and FGL2 was investigated by Dual Luciferase reporter (F) and RIP (G) in 293T cells.

For the fundamental investigation, the TargetScan database was used to predict the target genes of miR-146b, which was intersected with differentially upregulated genes in IBD from GSE42911 dataset. CYCS, POLR2F, RAB32, MPEG1, FGL2, SRI, COA5, and FOS were harvested (Fig. 3A). Among them, FGL2 could be significantly suppressed by miR-146b (Fig. 3B). In addition, it was found that FGL2 was overexpressed in the colon tissues of IBD mice and LPS-induced macrophages (Fig. 3 C and D).

Subsequently, the authors constructed miR-146b-overexpressed or miR-146b-depleted macrophages, and it was found that FGL2 expression could be efficiently enhanced by miR-146b depletion, but could be decreased by miR-146b overexpression (Fig. 3E) For further validation, Dual-Luciferase reporter assay disclosed that miR-146b mimics efficiently resulted in the decline of luciferase activity in FGL2-WT, while that in the FGL2-MUT group was not affected (Fig. 3F). Additionally, the RIP assay unraveled that miR-146b mimics remarkably caused FGL2 abundance in AGO2 antibody, in comparison with that in IgG antibody (Fig. 3G). In summary, the present results demonstrated that miR-146b could repress FGL2 in IBD-derived macrophages.

### FGL2 activated NF-κB and MAPK signaling pathways in LPS-induced macrophages

**Fig. 4:**
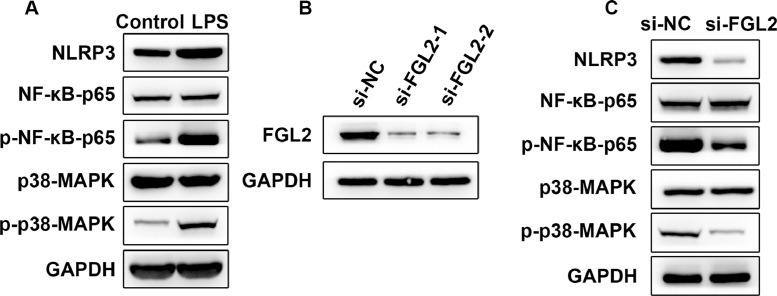
FGL2 activated NF-κB and MAPK signaling pathways in LPS-induced macrophages. NF-κB and MAPK pathways in LPS-induced macrophages (A), the transfection efficacy of FGL2 knockdown (B) and the phosphorylation level of p-NF-κB-p65 and p-p38-MAPK (C) were determined by Western blotting.

Considering the significant contribution of NF-κB and MAPK signaling pathways in macrophage polarization, the authors detected the expression of inflammasomes NLRP3, NF-κB-p65 and p38-MAPK in LPS-induced macrophages. Obviously, compared with the Control group, LPS resulted in the activation of NLRP3, NF-κB-p65 and p38-MAPK (Fig. 4A). Next, the authors knocked down FGL2 in macrophages (Fig. 4B), and it was found that FGL2 depletion considerably suppressed the expression of NLRP3, and the phosphorylation level of p-NF-κB-p65 and p-p38-MAPK (Fig. 4C), indicating that FGL2 could activate NF-κB and MAPK signaling pathways in LPS-induced macrophages.

### miR-146b suppresses inflammation and M1 polarization via inhibiting FGL2 in macrophages

**Fig. 5:**
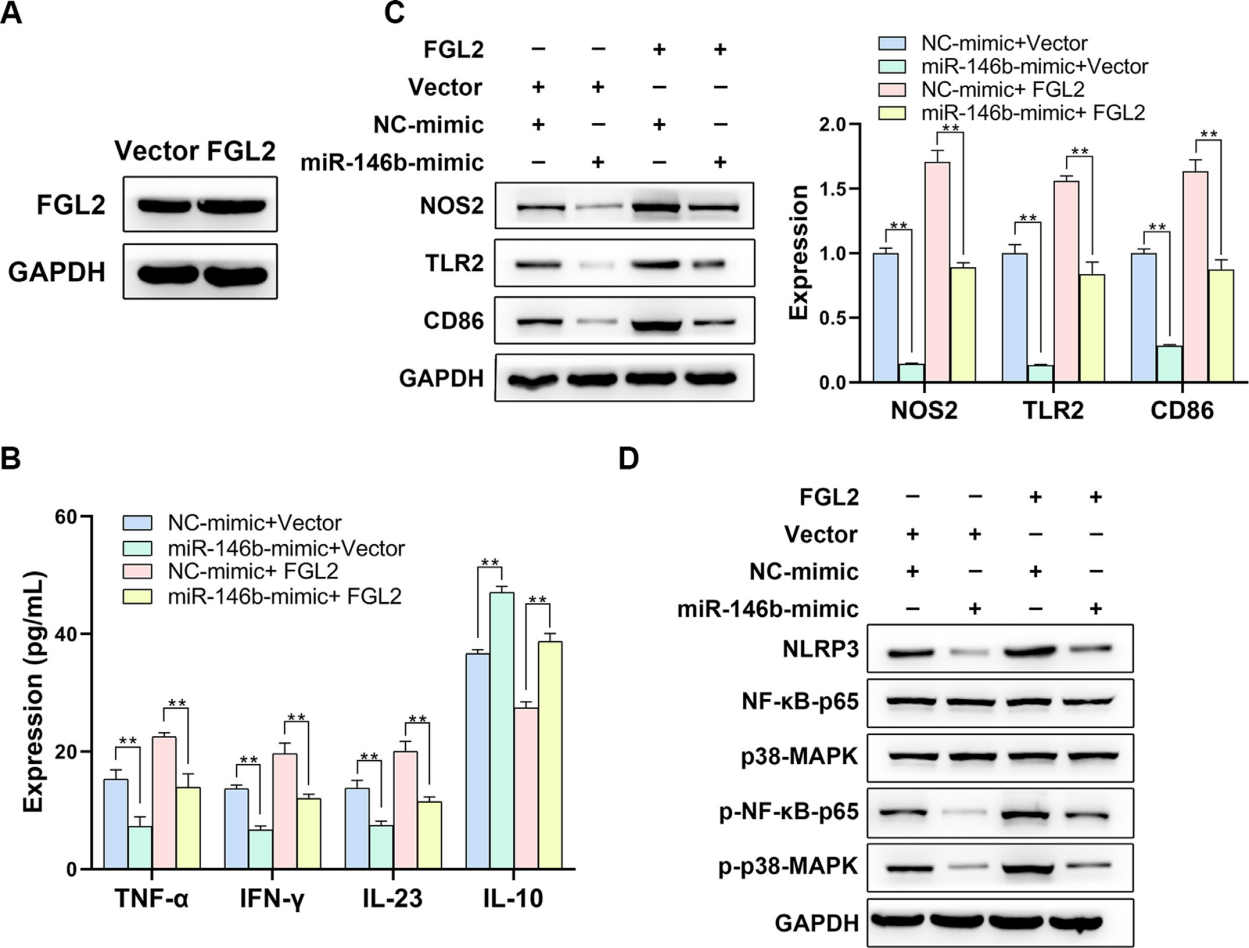
miR-146b suppresses inflammation and M1 polarization via inhibiting FGL2 in macrophages. miR-146b mimics and FGL2 overexpression plasmids were co-transfected into LPS-induced macrophages, and the transfection efficacy of FGL2 overexpression was examined by Western blotting (A). The levels of inflammation-related factors were determined by ELISA assay (B), and M1 macrophage polarization-related indicators (C) and the NF-κB and MAPK pathways (D) were determined by Western blotting.

To confirm whether miR-146b acts as an inhibitor for the inflammatory response in an FGL2-dependent manner, miR-146b mimics and FGL2 overexpression plasmids were co-transfected into LPS-induced macrophages (Fig. 5A). ELISA assay showed that miR-146b overexpression-induced loss of TNF-α, IFN-γ and IL-23 could be retained by FGL2 overexpression (Fig. 5B). Consistently, overexpressed FGL2 also played a recovery role in miR-146b overexpression-induced reduction of NOS2, TLR2, and CD86 (Fig. 5C). In addition, Western blotting exhibited that upregulated miR-146b restricted the expression of NLRP3, and the phosphorylation levels of p-NF-κB-p65 and p-p38-MAPK, which could be rescued by FGL2 overexpression (Fig. 5D). Overall, it was validated that miR-146b could exert inhibitory effects upon inflammation phenotype and M1 macrophage polarization through targeting FGL2.

### miR-146b ameliorates the inflammation phenotypes and M1 macrophage polarization of IBD mice in vivo

**Fig. 6:**
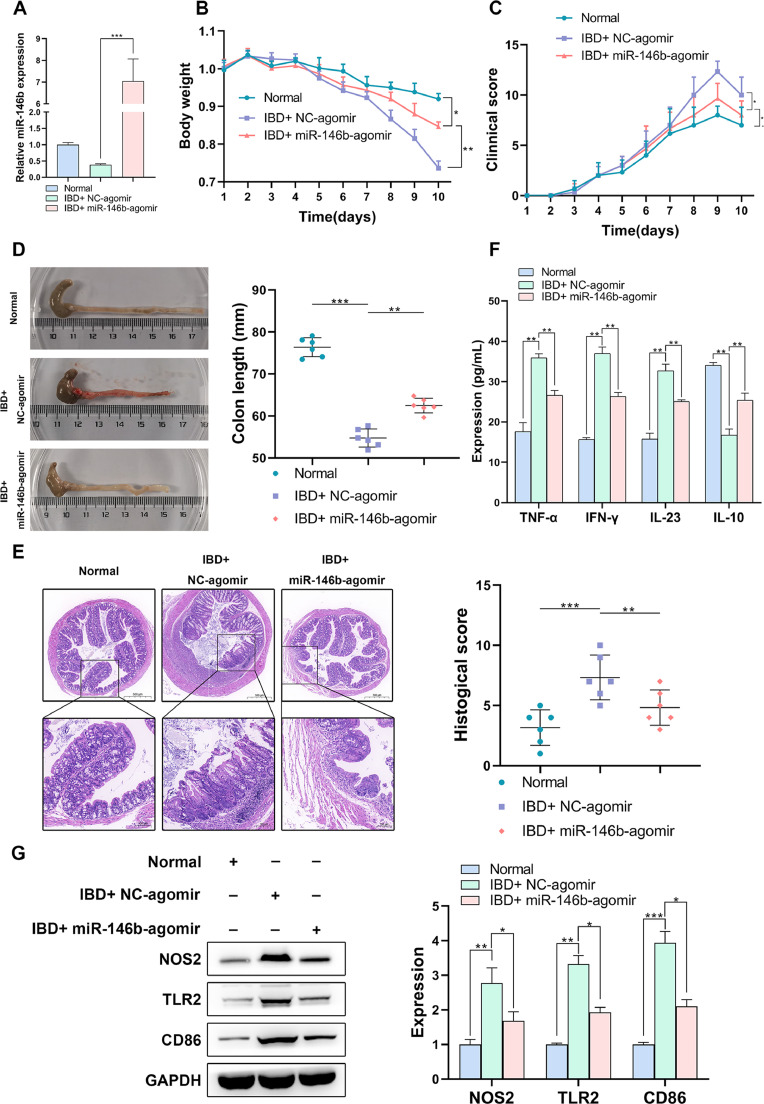
miR-146b ameliorates the inflammation phenotypes and M1 macrophage polarization of IBD mice *in vivo*. MiR-146b agomir was injected through the tail vein into IBD mice, and miR-146b expression in colon tissues was determined by q-PCR (A). The body weight (B), DAI scores (C), colon length (D) and the pathological changes (E) in IBD mice. The levels of inflammation-related factors were detected by ELISA assay (F), and M1 macrophage polarization-related indicators were determined by Western blotting (G).

To further validate the functional influence of miR-146b in IBD mice *in vivo*, lentiviral plasmids carrying miR-146b agomir were injected through the tail vein into IBD mice, and the expression of miR-146b in colon tissues was examined (Fig. 6A). Based on the changes of weight and DAI scores, it was noticed that miR-146b-agomir clearly increased the body weight of IBD mice, compared with mice injected with NC-agomir (Fig. 6B), but resulted in a drop in DAI scores (Fig. 6C). In addition, miR-146b overexpression significantly alleviated the length loss of the colon (Fig. 6D) and reduced the DSS-induced inflammatory cell infiltration (Fig. 6E). Furthermore, IBD mice injected with lentiviral plasmids carrying miR-146b agomir showed lower levels of pro-inflammatory cytokines like TNF-α, IFN-γ and IL-23 (Fig. 6F), and biomarkers (NOS2, TLR2 and CD86) concerning M1 macrophage polarization, in comparison with NC-agomir group (Fig. 6G). These data further validated the positive role of miR-146b in ameliorating the intestinal injury, inflammation phenotype and M1 macrophage polarization of IBD mice *in vivo*.

## Discussion

IBD is a non-specific chronic intestinal inflammatory disease, and the onset and development of IBD are generally accompanied by inflammatory response and injury in ileum, rectum and colon [22]. In this study, DSS-induced IBD mice models were constructed, and it was found that miR-146b inhibited the inflammatory phenotype and M1 macrophage polarization both *in vitro* and *in vivo*. In addition, FGL2 was identified as the target of miR-146b in IBD.

Increasing evidence has highlighted miRNAs as the indispensable regulator in IBD development 13, 14, 15 miR-146b has been previously reported closed related to IBD development [23,24]. Deng et al [23] have identified miR-146b gradually increased during mucosal regeneration in a murine colitis model. In a DSS-induced IBD mice, Deng detected miR-146b expression post DSS treatment. miR-146b expression significantly increased at day 3 post-DSS along with the reduced mucosal ulcers and extended PCNA+ epithelial cells. These results were different from the reduced expression of miR-146 in this study. The authors speculated miR-146b might be a positive regulator in IBD repairment. When the authors examined miR-146 levels in a DSS persistently treatment model, which made the intestinal mucosa in a damaged stage and the miR-146 has not been triggered by the immunity system. This result was also demonstrated in Chen's research, in which Chen found miR-146b was higher expressed in patients with Crohn's disease and ulcerative colitis [24] The immunity system was active in Patients with Intestinal mucosa injury, which might increase miR-146b to enhance the regeneration of Intestinal mucosa. Consistent with the present results, MTC-miR-146 mimics oral administration could protect the intestinal mucosa from DSS-induced injury. The authors injected lentiviral plasmids carrying miR-146b agomir through the tail vein into IBD mice and found miR-146b overexpression significantly ameliorated the symptoms and pathological changes in IBD injury, which could be specifically visualized in the alleviation of colon length loss and body weight of IBD mice, and the reduction of inflammatory cell infiltration, pro-inflammatory cytokines, and M1 phenotypes.

In a Mechanistic investigation, miR-146b overexpression relieved the inflammation levels and prevented M1 macrophage polarization, which was indicated by the downregulated proinflammatory cytokines (TNF-α, IFN-γ and IL-23), and M1 macrophages polarization markers NOS2, TLR2 and CD86. As we all known, M1 macrophages primarily produce pro-inflammatory cytokines and are involved in many chronic inflammatory diseases [25] The authors hypothesized that miR-146b may participate in regulating M1 phenotypes through inhibiting target genes. FGL2 was identified as the target of miR-146b via bioinformatics analysis and various methods, such as Dual Luciferase reporter and RIP assays. In addition, it was found that FGL2 could induce activation of NF-κB and MAPK signaling pathways in LPS-induced macrophages, which have been proved to be activated in IBD [26] Moreover, miR-146b and FGL2 were overexpressed in macrophages, and it was found that miR-146b overexpression reduced inflammation and M1 phenotypes of macrophages, which was rescued by FGL2 upregulation, suggesting that miR-146b acted as an inhibitor in inflammation response and M1 macrophage polarization in an FGL2-dependent manner. Apart from the present results, He et al. [27] suggested miR-146b could block the activation of M1 macrophage by targeting Stat1 in hepatic schistosomiasis, supplementing the evidence of miR-146b in macrophage polarization regulation.

Collectively, the inhibitory role of miR-146b was confirmed in the inflammatory response and M1 polarization in macrophages and in IBD mice. Furthermore, FGL2 was identified as the target gene of miR-146b, and miR-146b ameliorated the symptoms and pathological injury of IBD via inhibiting FGL2. The present study's findings may be regarded as novel evidence to relieve the inflammation response and injury of IBD.

## Authors' contributions

Yang Pan: Conceptualization; Data curation; Formal analysis; Investigation; Validation; Writing – original draft. Dan Wang: Conceptualization; Data curation; Formal analysis; Investigation; Validation; Writing – original draft. Fan Liu: Conceptualization; Writing – review & editing.

## Funding

This research received no specific grant from any funding agency in the public, commercial, or not-for-profit sectors.

## Conflicts of interest

The authors declare no conflicts of interest.

## Appendix Supplementary materials

Image, application 1
