# Supplementary material for: miR-146b suppresses LPS-induced M1 macrophage polarization via inhibiting the FGL2-activated NF-κB/MAPK signaling pathway in inflammatory bowel disease
Source: Clinics (Sao Paulo). 2022 Jun 21;77:100069. doi: 10.1016/j.clinsp.2022.100069 (PMC9234609; doi:10.1016/j.clinsp.2022.100069)
Supplement: Supplementary file 1 [file mmc1.pdf]

CLINICS-2021-3688_Supplementary Material

Supplementary Table 1 Sequences of siRNA against specific targets.

si-FGL2-1	5'-3'	aacaatgaaacagaggaaattaa
si-FGL2-2	5'-3'	gtgataacagagttagagaatta

Supplementary Table 2 Sequences of PCR primers used in this study.

GAPDH	Forward (5'-3')	TGTGGGCATCAATGGATTTGG
	Reverse (5'-3')	ACACCATGTATTCCGGGTCAAT
miR-146b	Forward (5'-3')	TGACCCATCCTGGGCCTCAA
	Reverse (5'-3')	CCAGTGGGCAAGATGTGGGCC
FGL2	Forward (5'-3')	GCCAAATGTGAGTCCCTGGAA
	Reverse (5'-3')	TTCCACCCAAGAGCACGTTTAAG
